# Potential Arbovirus Emergence and Implications for the United Kingdom

**DOI:** 10.3201/eid1204.051010

**Published:** 2006-04

**Authors:** Ernest Andrew Gould, Stephen Higgs, Alan Buckley, Tamara Sergeevna Gritsun

**Affiliations:** * Centre for Ecology and Hydrology Oxford, Oxford, United Kingdom;; † University of Texas Medical Branch, Galveston, Texas, USA

**Keywords:** Arbovirus, Emergence, United Kingdom, Risk

## Abstract

Arboviruses have evolved a number of strategies to survive environmental challenges. This review examines the factors that may determine arbovirus emergence, provides examples of arboviruses that have emerged into new habitats, reviews the arbovirus situation in western Europe in detail, discusses potential arthropod vectors, and attempts to predict the risk for arbovirus emergence in the United Kingdom. We conclude that climate change is probably the most important requirement for the emergence of arthropodborne diseases such as dengue fever, yellow fever, Rift Valley fever, Japanese encephalitis, Crimean-Congo hemorrhagic fever, bluetongue, and African horse sickness in the United Kingdom. While other arboviruses, such as West Nile virus, Sindbis virus, Tahyna virus, and Louping ill virus, apparently circulate in the United Kingdom, they do not appear to present an imminent threat to humans or animals.

## Abstract 2

Climate change can cause arthropodborne diseases to emerge.

This page was updated on July 19, 2006 to incorporate the corrections in Vol. 12, No. 8

More than 550 arboviruses have been identified (1). Many are mammalian and avian pathogens, 4 of which (West Nile virus [WNV], Usutu virus, Sindbis virus [SINV], and Tahyna virus) circulate in the United Kingdom in resident and migratory birds (2,3). The only zoonotic arboviruses isolated from field material in the United Kingdom are the flavivirus Louping ill virus (LIV) (4) and the bunyavirus Uukuniemi virus (5). LIV is included in the tickborne encephalitis virus (TBEV) complex that includes Kyasanur Forest disease virus and Alkhurma virus. These viruses can cause encephalitis and hemorrhagic disease (6). Other human pathogens in the genus *Flavivirus* that will be discussed in this review include yellow fever virus (YFV), dengue virus (DENV), Israel turkey meningoencephalomyelitis virus, and Bagaza virus. European human and animal pathogens in the genus *Alphavirus* include SINV, Ockelbo virus, and Chikungunya virus and in the family *Bunyaviridae*, sandfly fever Naples virus (often referred to as Toscana virus), sandfly fever Sicilian virus, Crimean-Congo hemorrhagic fever virus (CCHFV), Inkoo virus, and Tahyna virus, which is widespread throughout Europe. Rift Valley fever virus (RVFV) and Nairobi sheep disease virus (NSDV) could be introduced to Europe from Africa through animal transportation. Finally, the family *Reoviridae* contains a variety of animal arbovirus pathogens, including bluetongue virus, which is currently circulating in Europe, and African horse sickness virus, which has been found in Europe but is not currently circulating. This review considers whether any of these pathogenic arboviruses are likely to emerge and cause disease in the United Kingdom in the foreseeable future.

## Factors That May Determine Arbovirus Emergence

Transmission of arboviruses between invertebrates and vertebrates imposes constraints on evolution and dispersal, which are reflected in their phylogenetic relationships (7). However, the influence of modern life on arbovirus emergence cannot be overemphasized. The following human activities may influence arbovirus emergence: 1) increased transportation of animals, humans, plants, arthropods, and other materials; 2) increased outdoor leisure activities; 3) reduced or nonexistent arthropod control programs; 4) deforestation; 5) reforestation; 6) land reclamation; 7) altered farming practices; 8) urbanization programs; 9) irrigation projects, including building dams or creating reservoirs and lakes; 10) military activities; 11) movement of military personnel and local populations in war zones; 12) natural disasters, such as flooding; and 13) the early effects of climate change (8).

## Examples of Emerging Tickborne Arboviruses

TBEV complex viruses rarely cause disease in indigenous forest animals but may emerge as pathogens when they infect introduced species. Members of the complex have evolved and dispersed westward across Asian and European forests during the past 3–5 millenia. This dispersion is driven by *Ixodes* spp. vectors that inhabit the moist forest undergrowth. Nymphal ticks may infect newly hatched larvae when they co-feed on forest animals. Since these vertebrates do not become sick or have detectable viremia, this direct method of vector infection is known as nonviremic transmission. In contrast, LIV and related viruses in Spain, Turkey, Greece, and nearby regions produce viremia and fatal encephalomyelitis in domesticated animals when infectious ticks feed on them (9). These animals have no genetic resistance or immunity to the TBEV-related viruses that have emerged from the forests (8).

Kyasanur Forest disease virus emerged in Karnataka, India, in 1957 after forests were cleared for urbanization and farmland reclamation. Arboreal monkeys scavenging on the exposed forest floor became infested with Kyasanur Forest disease virus–infected ticks from the undergrowth. Local inhabitants and veterinary scientists who examined dead and dying monkeys also became infected. A closely related virus, Alkhurma virus, emerged in Saudi Arabia in 1992, causing hemorrhagic disease in butchers who handled sheep imported into Saudi Arabia for the Hajj (10). Alkhurma virus was detected in ticks removed from imported sheep, but the country from which these sheep originated has not been identified.

CCHFV emerges sporadically in Africa, Asia, and Europe. This arbovirus may be transmitted to animals by ticks, especially members of the *Hyalomma* genus. Emergence of CCHFV largely depends on the transportation of livestock such as cattle and goats, on which infected ticks feed. Most cases of hemorrhagic disease occur in humans involved with the livestock industry. Since >21 tick species are present in the United Kingdom, and since viruses such as CCHFV can infect a wide variety of ticks, the introduction and emergence of CCHFV are not inconceivable.

NSDV (family *Bunyaviridae*, genus *Nairovirus*) is transmitted to sheep by *Rhipicephalus appendiculatus* and causes fever and gastroenteritis; the infection is often fatal. NSDV is distributed widely throughout Africa (1) but not elsewhere. However, the closely related Ganjam virus is found in India, which may imply that NSDV has been transported in ticks on animals imported to India from Africa. Both NSDV and Ganjam virus produce febrile illness with polyarthritis in humans.

## Examples of Emerging Mosquitoborne Arboviruses

YFV circulates among simians and *Aedes* and *Hemagogus* spp. mosquitoes in the forest canopy and adjacent savannah regions of Africa and South America. In Africa, nonhuman primates do not become ill from YFV infection, presumably because the virus has adapted to the simian hosts. Nevertheless, human yellow fever epidemics do occur in Africa because the virus emerges sporadically from the jungles and savannah into rural and urban areas, where most of the human population is susceptible. In South American jungles, moribund monkeys are usually found immediately before local epidemics of yellow fever, implying that the virus recently encroached into the region. YFV was introduced into South America on ships that sailed from Africa during the past few centuries (8). Many European and North American ports observed cases of yellow fever. In some cases, the virus was carried on sailing ships from Cuba to Europe, causing localized outbreaks where the ships docked (11). Infections in Swansea (Wales) and St. Nazaire (France) resulted from the bite of *Aedes aegypti* mosquitoes released from the newly arrived ships. However, the disease immediately disappeared when the local temperature dropped and the tropical mosquitoes died. Thus, such viruses are unlikely to emerge in the United Kingdom.

The 4 DENV serotypes emerged more recently than YFV (8) and can generate epidemics in humans without depending on a sylvatic cycle in the jungles. Therefore, in contrast to YFV, which is maintained in the jungles of Africa and South America, DENV continues to be dispersed by humans and mosquitoes throughout the tropics and subtropics worldwide (8,12). Presumably, as *Ae. aegypti* and *Ae. albopictus* continue to spread, the geographic distribution of these viruses will also expand.

Since birds are highly mobile and can travel great distances in a relatively short time, they play a role in arbovirus dispersal. SINV, for example, is carried annually between South Africa and Scandinavia by migrating birds (12). SINV is rarely associated with disease in Africa. However, when cases of arthritis, polyarthritis, and fever associated with rash were recorded in Scandinavia, the antigenically closely related Ockelbo virus was isolated. Presumably, Ockelbo virus evolved from introduced SINV and subsequently became established in a local transmission cycle. In the New World, St. Louis encephalitis virus is also carried long distances by migrating birds. It was probably introduced into South America from Africa during the past few centuries and gradually dispersed by migrating birds to North America (13). More recently, WNV was introduced into the New York area, probably in 1999. The virus became established in birds and was rapidly dispersed north to Canada; south to Florida, Mexico, and the Caribbean; and west, reaching California in 2004 (http://www.cdc.gov/ncidod/dvbid/westnile/). The WNV that was introduced into New York was related to an Egypt/Israel strain that is also present in the Volga River Delta. Despite its high virulence in birds (particularly corvids), horses, and alligators in North America, WNV circulates relatively harmlessly in the Old World, presumably reflecting co-evolution of virus and host over a long period.

Other emerging flaviviruses include Usutu virus, which appeared unexpectedly in Austria in 2000 (14), killing birds in Vienna and surrounding areas. Previously, Usutu virus had only been found in Africa. Israel turkey meningoencephalitis virus, isolated in Israel, is phylogenetically closely related to the African Bagaza virus (7). Thus, Israel turkey meningoencephalitis virus may represent the birdborne version of Bagaza virus.

RVFV (family *Bunyaviridae*, genus *Phlebovirus*) is usually transmitted by *Aedes* spp. mosquitoes and occasionally infects livestock, producing epizootics with high death rates in sheep and other ruminants. Humans in areas where RVFV is epizootic may also become infected. Until 2000, RVFV was most commonly found only in eastern, western, and southern Africa. However, in September 2000, an outbreak was reported in Saudi Arabia and subsequently in Yemen, representing the first cases identified outside Africa and showing the potential for global dispersal.

Bluetongue virus (family *Reoviridae*, genus *Orbivirus*) is transmitted to domestic and wild ruminants by culicoide midges. Bluetongue virus epidemics can threaten entire livestock populations and were first described in South Africa after Merino sheep were introduced from Europe in the late 18th century. The virus subsequently dispersed globally, recently reemerging in Italy, Spain, and Portugal (15), most likely through the transportation of ruminants. Whether bluetongue virus will emerge in the United Kingdom likely depends on the effects of climate change on vector dispersal.

## Arboviruses Most Likely To Circulate in the United Kingdom

The English Channel and North Sea are not barriers to migrant birds, bats, or commercial transportation systems. Therefore, most mainland European arboviruses could be introduced and established in the United Kingdom. The Table presents a summary of all recognized zoonotic mosquitoborne and tickborne arboviruses circulating in western Europe. While Uukuniemi virus has not been shown to be a human pathogen, it circulates widely in Europe, and seropositivity in humans has been demonstrated many times. YFV, DENV, RVFV, Ganjam virus, and NSDV are excluded from the Table because they only arise as imported viruses. Only LIV and Uukuniemi virus have been isolated from field specimens in the United Kingdom, probably because of limited investigation, although the other viruses in the Table have been isolated in Europe. Serologic evidence obtained from bird sera (2) implies that at least 3 additional arboviruses, WNV, Usutu virus, and SINV, circulate in the United Kingdom. Currently, TBEV has not dispersed west beyond the Alsace forests in France (29). LIV, Spanish sheep encephalomyelitis virus, and Greek goat encephalomyelitis virus, on the other hand, represent more recent lineages of western European and Far Eastern TBEV that have emerged on the moorlands after the introduction of sheep and goats for grazing (8).

**Table:** Zoonotic arboviruses identified in western Europe (not including Russia) by serologic testing or isolation*

Country	Seropositive†	Virus isolation	Reference
Sweden	SINV, TBEV, INKV, BATV	SINV, INKV, BATV	(12,16)
Finland	SINV, TBEV, TAHV, INKV, BATV	TBEV, INKV, BATV, UUKV	(12)
Norway	LIV, TBEV, TAHV, INKV, BATV	SINV, LIV, TBEV, INKV, BATV, UUKV	(12,17)
Poland	SINV, WNV, TBEV, UUKV	UUKV	(12,18)
Estonia		SINV	(12)
Austria	SINV, SFV, CHIKV, TBEV, USUV, TAHV, BATV	TBEV, USUV, TAHV, BATV	(12,19)
Czechoslovakia	SINV, WNV, TBEV, TAHV, BATV, LEDV	SINV, WNV, TBEV, TAHV, BATV, UUKV	(12,20)
France	TBEV, WNV, TAHV	TBEV, WNV, TAHV	(12,21)
Germany	TBEV, TAHV, BATV	TBEV, TAHV, BATV	(12)
Portugal	SINV, CHIKV, WNV, TAHV, BATV, TBEV	WNV	(12,22,23)
Spain	SFV, CHIKV, TBEV, SSEV, TAHV		(12,24)
Italy/Sicily	SINV, CHIKV, WNV, TBEV, TAHV, SFSV	SINV, WNV, TBEV, SFSV, TAHV	(12,25)
Yugoslavia	SINV, TBEV, TAHV, BATV	TBEV, TAHV, BATV	(25–27)
Albania	TBEV, TAHV		(12)
Hungary	WNV, TAHV	WNV, TAHV, UUKV	(20,25)
Romania	SINV, SFV, WNV, TBEV	TBEV, WNV, TAHV, BATV, LEDV	(12,17,27)
Greece	SINV, TBEV, TAHV, GGEV, CCHFV	CCHFV	(12,28)
United Kingdom/Ireland	LIV, SINV, WNV, USUV, TAHV, UUKV	LIV, UUKV	(2,5)

*Adapted and modified from Lundstrom (12), which contains a more comprehensive list of relevant references. SINV, Sindbis virus; TBEV, tickborne encephalitis virus; INKV, Inkoo virus; BATV, Batai virus; TAHV, Tahyna virus; UUKV, Uukuniemi virus; WNV, West Nile virus; SFV, Semliki Forest virus; CHIKV, Chikungunya virus; USUV, Usutu virus; LEDV, Lednice virus; SSEV, Spanish sheep encephalomyelitis virus; SFSV, sandfly fever Sicilian virus; GGEV, Greek goat encephalomyelitis virus; CCHFV, Crimean-Congo hemorrhagic fever virus; LIV, Louping ill virus. †Positive reactions to dengue virus or yellow fever virus are not included as they are assumed to represent cross-reactions with WNV or other Japanese encephalitis complex flaviviruses.

Three alphaviruses are listed in the Table, SINV, Semliki Forest virus, and Chikungunya virus. SINV has been isolated from field material in Scandinavia, Estonia, Czechoslovakia, and Italy. This virus is widespread throughout the Old World. In Albania, seroconversion to Chikungunya virus was detected in 5 patients with mild flulike symptoms (12); other findings of antibodies to Chikungunya virus have been reported, but evidence of cross-reactivity with other alphaviruses was not always tested. Among the remaining zoonotic arboviruses included in the Table, Tahyna virus is the most common. This virus is widespread throughout western Europe in humans and many nonhuman mammalian species and birds (12). Inkoo virus has only been found in Scandinavia, but few investigations have taken place elsewhere. While investigations for Batai virus have not been extensive, this virus is also widespread throughout western Europe (12).

## Probability That Arboviruses Will Emerge as Epidemic Pathogens

### Tickborne Arboviruses

Many arboviruses emerged relatively recently (30) and have therefore had little time to be dispersed to new environments, such as the United Kingdom. Although birds carry millions of ticks north and south annually between Africa and Europe, tickborne African bunyaviruses and flaviviruses have not yet appeared as notable pathogens in the United Kingdom. European, Siberian, and Far Eastern strains of TBEV have been isolated in Estonia, probably introduced by migratory birds (31). However, they have not caused substantial disease in these regions. TBEV is less virulent than LIV for sheep (6) and might, therefore, become less dominant than LIV if it were introduced into the sheep-grazing moorlands of the United Kingdom. Whether such introduced viruses would emerge may reflect the level of contact between humans and farm animals. Although ticks are less susceptible to the effects of environmental disruption, the specific region into which TBEV-carrying ticks are introduced might determine whether they survived and then reproduced. The United Kingdom has an abundant supply of *Ixodes* ticks, but the risk that virulent Far Eastern TBEV would be introduced, become established, and emerge as an endemic pathogen in UK forests is probably low. Alternatively, in the case of CCHFV, the fact that this virus has been isolated from ticks in Greece, the Balkans, and western Russia in the Volga Delta cannot be ignored. Other tickborne virus pathogens are presumably subject to the same qualifications.

### Mosquito-, Sandfly- and Midge-transmitted Arboviruses

When one takes into account the information in the Table and the serologic evidence of antibodies against SINV, WNV, Usutu virus, and Tahyna virus in sera of migrant and resident UK birds, the likelihood is high that mosquitoborne arboviruses, particularly those associated with ornithophilic mosquitoes, such as *Culex* spp., circulate, albeit harmlessly, in the United Kingdom. Other arboviruses circulating in Europe that are not recognized in the United Kingdom include Toscana virus, bluetongue virus, and African horse sickness virus, which was probably introduced into Spain after the importation of infected horses.

Tahyna virus, which causes encephalitis in humans, is widespread across western Europe (Table). Birds and rabbits are vertebrate hosts. Since the movement of rabbits between the United Kingdom and mainland Europe is extensive, and since many bird migratory routes from Africa pass over the United Kingdom, Scandinavia, and mainland western Europe, Tahyna virus is most likely to appear, if not emerge, in the United Kingdom.

Why has none of these viruses yet emerged as a noticeable pathogen in the United Kingdom? Perhaps they have, and we do not realize it. Recent reports state that <40% of human deaths from viral encephalitis are attributed to a specific etiologic agent in the United Kingdom (32). Moreover, no systematic studies have been conducted to determine the extent to which Tahyna virus is circulating, even though the virus is known to be present in UK birds (3). In addition, on the basis of serologic tests (2), a SINV-like virus also circulates among birds in the United Kingdom, possibly vectored by *Culex* mosquitoes. In Scandinavia, this virus causes rash, fever, and acute polyarthritis in humans.

### Necessary Arthropods for Arbovirus Emergence

At least 21 species of hard ticks and 3 argasids are recognized in mainland Britain and its islands (33). Most parasitize mammals, but at least 12 prefer birds, and 2 are regularly found on reptiles. In many parts of the United Kingdom, LIV has been isolated from *I. ricinus* ticks, which are believed to maintain LIV in the wild by nonviremic transmission during co-feeding (34). Other tick species, such as *Ornithodorus maritimus* and *I. uriae*, transmit bunyaviruses, nairoviruses, and orbiviruses among seabirds (5,35) that inhabit the cliffs around the UK coastline. However, these seabird-tickborne viruses do not cause disease in mammals in the United Kingdom. Nevertheless, the United Kingdom has a wide range of ticks that can transmit arboviruses.

At least 33 mosquito species are endemic in the United Kingdom (36), but their susceptibility to infection by arboviruses and ability to replicate and transmit them to vertebrate hosts have not been assessed. At least 9 of these mosquito species have been linked to transmission of WNV (37). Many related viruses cause encephalitic infections and are transmitted in nature between birds and mosquitoes, particularly *Culex* spp. Six of the 33 endemic mosquito species feed on humans and birds, which makes them potential bridge vectors (36). Eleven of the remaining 33 species bite humans. Clearly, the United Kingdom has a wide range of potential mosquito vectors. Moreover, if DENV vector species that survive in colder temperatures, such as *Ae. albopictus*, become established, the United Kingdom may be more vulnerable to outbreaks of dengue fever.

Several pathogenic mosquitoborne viruses have been isolated from animals, mosquitoes, and humans in Europe and Scandinavia (Table). The same viruses are likely also to exist in the United Kingdom, but why do these mosquitoborne viruses not cause disease? Possible explanations include the following: 1) the level of disease in the United Kingdom is low and therefore missed by surveillance systems; 2) the climate rarely satisfies the requirements for efficient arbovirus transmission by mosquitoes; 3) resident arthropods in the United Kingdom have low competence for arboviruses; 4) low-level immunity has been established among mammalian and avian species involved in virus circulation; and 5) viruses are circulating, but nonviremic transmission of the virus is occurring between mosquitoes co-feeding on nonsusceptible vertebrate hosts (38).

Some species of *Culicoides* (biting midges) are vectors of animal diseases, such as bluetongue virus (e.g., *Culicoides imicola* and *C. variipennis*) and African horse sickness. According to Campbell and Pelham-Clinton (39), 41 *Culicoides* spp. occur in the United Kingdom; dominant species include *C. impunctatus* in Scotland and *C. obsoletus* in southern England. In Scotland, 37 species occur, ≈20 of which are mammalophilic, but only 5 of which bite humans. In the highlands of Scotland, *C. impunctatus* can reach pest proportions and is associated with "nuisance biting." However, bluetongue virus and African horse sickness virus are not present in the United Kingdom, and because its climate is cooler than that of southern Europe, they are not likely to become established in the near future (http://www.defra.gov.uk/animalh/diseases/notifiable/disease/bluetongue.htm).

Sandflies are not known to be endemic in the United Kingdom, but they are present in all Mediterranean countries. As climate change takes effect, sandflies are expected to become established in the United Kingdom. Thus, sandfly fever/Toscana virus might emerge later in this century.

Finally, direct transmission of arboviruses between vertebrates (i.e., not requiring an arthropod vector) has been recognized for many years. For example, LIV and TBEV may be transmitted through the milk of sheep and goats (6). Moreover, evidence is accumulating that mosquitoborne arboviruses can be transmitted among vertebrate species by routes additional to those involving arthropods (40). While invertebrates almost certainly contribute to arbovirus transmission in northern Europe, the relatively lower numbers and density of mosquitoes in these regions might limit their efficiency in disease transmission. These alternative nonvectored modes of transmission could in part explain how such viruses might circulate between vertebrates in environments previously considered suboptimal for efficient circulation of arboviruses.

## Conclusion

Arboviruses rarely cause disease in their maintenance hosts or vectors; consequently, viremic blood is not available for vectorborne transmission. Arboviruses have therefore developed other transmission strategies to ensure their survival. For years, the favorite mechanisms to explain virus survival in the absence of disease were vertical transmission, overwintering, or persistent infection in the arthropod or vertebrate host. We now recognize that the process of nonviremic transmission between co-feeding ticks or co-feeding mosquitoes, and perhaps ticks co-feeding with mosquitoes, is a powerful force for virus transmission even in the presence of immunity. Therefore, this repertoire of transmission strategies can ensure arbovirus perpetuation in the absence of disease.

Armed with this versatility, arboviruses can adapt to virtually any situation. Most arboviruses have evolved a sylvatic existence that is infrequently associated with disease in vertebrate hosts. However, in the presence of susceptible hosts, an environment that favors rapid arthropod amplification, an efficient mechanism for dispersal, and a suitable climate for transmission, DENV, RVFV, Japanese encephalitis virus, and YFV may cause epidemic outbreaks of immense proportions.

This variety of critical factors must come together at an appropriate time for an arbovirus to emerge as an epidemic pathogen, a situation that has rarely happened in the United Kingdom. Of course, if climate change has the effects that experts predict it will, the situation could change during the 21st century. In conclusion, despite compelling evidence that potentially highly pathogenic arboviruses are circulating or, alternatively, are being introduced annually into the United Kingdom, no imminent threat exists that they will cause epidemic outbreaks of serious concern to public or environmental health.
