# Correction, Vol. 12, No. 4

**DOI:** 10.3201/eid1208.C11208

**Published:** 2006-08

**Keywords:** Correction, Ernest A. Gould,

In "Potential Arbovirus Emergence and Implications for the United Kingdom" by Ernest A. Gould et al., an error occurred. The first paragraph of the article incorrectly states that African horse sickness virus is circulating in Europe. The sentence should read "Finally, the family *Reoviridae* contains a variety of animal arbovirus pathogens, including bluetongue virus, which is currently circulating in Europe, and African horse sickness virus, which has been found in Europe but is not currently circulating."

The corrected text appears in the online article at http://wwwnc.cdc.gov/eid/article/12/4/05-1010_article.htm.

